# How Colposcopy Misses Invasive Cervical Cancer: A Case Report from the IMPROVE-COLPO Study

**DOI:** 10.1155/2016/5857370

**Published:** 2016-10-18

**Authors:** Jeff Livingston, Emmanouil Papagiannakis

**Affiliations:** ^1^MacArthur OB/GYN, Irving, TX, USA; ^2^DySIS Medical Inc, Tampa, FL, USA

## Abstract

Colposcopy with biopsy is pivotal to cervical cancer prevention programs as it is called to identify the precancerous lesions on patients that screen positive. We present a cervical cancer case from IMPROVE-COLPO, a postmarketing, multicenter, two-arm observational study on US community colposcopy that collects outcomes with the adjunctive Dynamic Spectral Imaging (DSI) in its prospective arm. A 45-year-old woman was seen for suffering of heavy periods. She had cytology of Atypical Squamous Cells of Undetermined Significance (ASC-US) and was Human Papillomavirus (HPV) positive. Her colposcopy did not recognize the underlying condition and opted for no biopsy. The DSI assessment led to a biopsy of a lesion challenging traditional colposcopic templates: small, away from the cervical os, with slow acetowhitening development. Pathology review revealed the presence of invasive squamous carcinoma. In the era of sensitive screening, it is concerning that invasive cancers can challenge colposcopy and that the way to improve colposcopy is to collect multiple biopsies from each patient. The case presented indicates that the adjunctive objective assessment by DSI increases reassurance that observations outside of traditional standard visual templates are not underestimated or ignored.

## 1. Introduction

Cervical cancer in the US is prevented by rigorous screening that halved incidence and mortality over the last decades. However, it remains a considerable burden, with 12,990 cases and 4,120 deaths expected in 2016 [1]. Guidelines were updated recently [2, 3] to improve the age-appropriate strategies with implementation of Human Papillomavirus (HPV) testing and extend the screening intervals. This triggered concerns about increased cancer risks compared to annual Pap testing [4] and has not been widely adopted as it is a paradigm shift and the different possible clinical pathways are less clear for healthcare providers and patients than the “get your annual pap” approach [5]. Colposcopy is pivotal for managing women that test positive and is responsible for identifying and diagnosing disease, by selective tissue sampling for histological confirmation. It is a subjective examination that balances the visual and empirical assessment of the cervix, biopsy decisions, and placement selection, with patient experience and associated costs. Colposcopic practice is nonstandardized and large studies have demonstrated that it misses important disease [6, 7]. It cannot be predicted at what rate missed disease, even Cervical Intraepithelial Neoplasia grade 3 (CIN3), would eventually become invasive, as the risk accumulates over time. Therefore, colposcopy on any given patient could prove critical as her risk is unpredictable. Colposcopy has been relying on frequent patient screening, but in view of the three- to five-year intervals [2], the adequacy of colposcopy, especially in the community setting, where it is often practiced by generalists, to identify invasive disease needs assessment.

The IMPROVE-COLPO study is an observational, two-arm colposcopy study recruiting women according to current guidelines and common practice [3] across multiple facilities in the USA. The study was approved by a central Institutional Review Board (IRB) (E&I Review Services, Corte Madera, CA, USA). The study collects pragmatic outcomes of routine colposcopic practice after the adoption of a new commercial digital colposcope (DySIS, DySIS Medical, Tampa, FL) which integrates a method for quantifying and mapping the cervical acetowhitening, Dynamic Spectral Imaging (DSI). The DSI describes the acetowhitening with a color-coded map. This map is calculated by quantifying the acetowhitening onset, intensity, and persistence and is used adjunctively to the colposcopic examination, supporting assessments, biopsy placement, and documentation. Previous work showed that DSI improves the sensitivity for patients with high-grade CIN [8]. We present and discuss a case of invasive cervical cancer identified with a DSI-guided punch biopsy.

## 2. Case Presentation

A 45-year-old non-Hispanic Caucasian patient, G3P3, nonsmoker, had a gynecologic evaluation after experiencing heavy periods for the last five years with increasing pain. She had no relevant history and her previous Pap test, a year earlier, had been normal. Pelvic ultrasound showed a normal sized uterus with normal appearing right ovary that had a simple cyst of 3.7 cm and a 1.1 cm endometrium. Her cervical screening test was Atypical Squamous Cells of Undetermined Significance (ASC-US), Human Papillomavirus (HPV) positive, so she underwent colposcopy. She provided signed consent to be recruited in the study.

**Figure 1:**
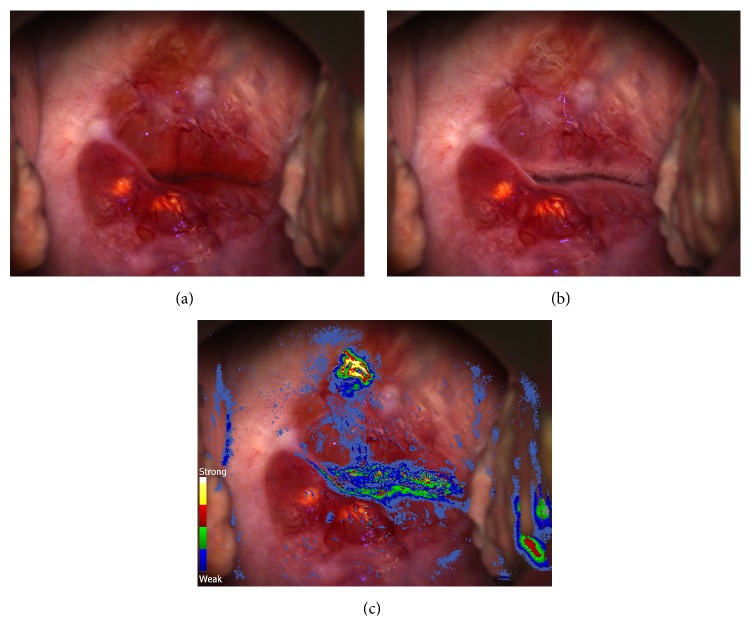
(a) The cervix prior to the application of acetic acid; (b) the cervix 30 seconds after acetic acid was applied. (c) The DSI color-coded map, overlaid on the cervix. The upper part of the DSI color scale (red, yellow, and white) indicates persistent/intense acetowhitening. The 11-12 o'clock area highlighted by the map was biopsied and invasive squamous carcinoma was found.

During colposcopy, the transformation zone of the cervix, including the outer limits, was entirely visualized (Figure 1(a)) and acetic acid (3%) was applied. No significant findings were observed, except for Nabothian cysts at 6 and 8 o'clock. Minimal acetowhitening reaction was observed around the external os (Figure 1(b)). The assessment was that there were no lesions suspected for neoplasia and biopsy was not warranted, in accordance with guidelines [3]. Subsequently, the DSI color-coded map was revealed and reviewed (Figure 1(c)). It highlighted a small acetowhite area around 11-12 o'clock, with colors representing strong/persistent acetowhitening on the color scale of the map (included in Figure 1(c)). The standardized working distance and field of view of the colposcope allow the estimation of the lesion size (*post hoc*) to be approximately 0.43 cm in the horizontal direction. Prompted by the DSI indication, a punch biopsy was performed at that site. Based on the patient's condition, additional samples were collected from the endocervix and the endometrium.

The histopathological review of the punch biopsy showed an invasive squamous carcinoma that was undermining the mucosa and that the depth of the biopsy did not fully represent the full depth of the malignancy. The endocervical curettage was negative and the endometrial biopsy benign. The patient was referred for oncology consultation and had a PET/CT scan to stage the carcinoma as well as investigate whether there were any additional findings that could explain her lower abdominal discomfort other than the ovarian cyst. The PET/CT scan revealed some minimal reactive inguinal nodes, but no other evidence of metastatic disease. There was no significant contrast in the region of the cervix. The least-invasive approach of cone biopsy was discussed and suggested to the patient; however as she was not considering future pregnancies, she preferred and insisted on having the most definitive surgery. She underwent robotic laparoscopic radical hysterectomy with pelvic lymphadenectomy and bilateral salpingo-oophorectomy. The pathology review confirmed a poorly differentiated squamous cell carcinoma of the cervix, present in the 9:00 to 12:00 quadrant, 0.5 cm in greatest horizontal expansion, invasive 0.1 cm into the stroma. It also reported the presence of severe squamous dysplasia (CIN3). All margins were benign and negative for dysplasia. No other malignancies were identified, and the patient remains under oncologic surveillance.

## 3. Discussion

Cervical cancer is curable if identified early and is not expected in women who are screened appropriately. However, no test is perfect, and, as this case illustrates, some women fall through the safety nets. Cytology is not sensitive and HPV testing is sensitive but not specific to disease; their combined use stratifies patients according to risk and refers those needing further investigation [2, 3]. Patient history and demographics play a large role in colposcopic decision making, but it is mostly the referral cytology that affects decisions, especially when it is low-grade, as the chance of significant disease is then rather low [9]. The accuracy of colposcopy, largely a pattern recognition examination, is documented to be poor [6, 7], and even cervical cancers are underestimated at a significant rate [6]. Colposcopy can be challenged by disease that does not fall within specific templates developed by individual training and experience. It is important to notice that cervical cancer is a rare encounter during colposcopy of patients with LSIL or ASC-US cytology [9], especially in the US community setting that mostly serves a well-screened low-risk population, and may therefore not be recognized.

Nowadays' sensitive screening methods are likely to be picking up lesions comparatively earlier than before [9]. These lesions could be smaller, in contrast to the past when high-grade lesions were obvious by the time patients had colposcopy. Rooted in the era preceding nowadays' sensitive screening methods, colposcopy expects that lesion severity correlates well with lesion size [10]. Effectively, this can lead to underestimation of smaller lesions visualized at colposcopy, especially on women with lower-grade cytology, like in this case. However, the support for this argument does not come from prospective studies with end point of the potential progression of lesions of different sizes to cancer, but from retrospective data analysis based on 39 cases of microinvasive cancer identified in excisional treatments from the 1980s [11]. In those cases, the size of the microinvasion was compared to the size of coexisting CIN3 lesions on the cone specimen and not on its apparent colposcopic appearance. On the basis of that article, one cannot exclude that some lesions that may be underestimated at colposcopy as low-risk (and may not even be biopsied) due to an apparent small size may penetrate into the stroma without expanding in size. Therefore, rarely, but possibly, invasive carcinoma may originate from smaller lesions that would be harder to identify as suspicious at colposcopy [9]. Furthermore, colposcopy expects neoplasia to be at its more severe state closer to the new squamous-columnar junction and can be misled by observations away from the cervical os, considering them not worrisome.

Acetowhitening is the main test used at colposcopy to highlight and visualize abnormalities. It is a transient phenomenon with onset, intensity, and persistence that vary across the different areas of the cervix, and it can be challenging to assess it accurately, when observing through the colposcope, and potentially focusing attention to where abnormality is most likely to be. Its dynamics can be a challenge to visualize, assess, and interpret accurately. Often, one must wait and observe the entire cervix for a few minutes, to allow the acetowhitening to fully develop to, for example, differentiate the slow-onset/persistent from rapid/quickly-fading areas. Differentiation and comparison of acetowhitening at different parts of the cervix are important for effective and accurate biopsy placement. In the case presented here, it took two minutes for acetowhitening to reach its peak intensity at the site with the invasion, suggesting that assessments made sooner than this, not uncommon in routine colposcopy practice, could be misleading.

Colposcopy, the single and irreplaceable second-level procedure for triage of cervical abnormalities, is challenged to match the today's screening tests, especially in view of the extended intervals that increase the risks for patients with missed disease [4]. Apart from the importance of colposcopic training and quality assurance, there is a need for standardization in the way colposcopy is performed and the way that its observations are assessed, and the role that technology can play in this is recognized and anticipated [12].

The DSI method standardizes the methodology of observation and analysis of acetowhitening, independently of location on the cervix or patient history and referral. It complements a thorough colposcopic examination and supports assessments and placement of biopsies based on an objective standard [8]. This could be an alternative approach to performing generalized random, 4-quadrant [13], or multiple biopsies [14] on every patient. It must be noted that this case report is by no means sufficient to demonstrate the potential of the DSI method to improve colposcopic outcomes. Prospective trials have shown that DSI combined with a thorough colposcopic examination increases the sensitivity of identifying patients with high-grade disease significantly [8]. For this ASC-US/HPV patient, the use of the DSI map to guide her single biopsy after the colposcopist decided no biopsy was warranted led to the timely detection and treatment of an invasive squamous carcinoma that would have otherwise been missed with an obvious risk for further progression. One cannot argue whether another highly trained and experienced colposcopist would have identified this cervical cancer, as the subjectivity and interobserver variability of colposcopy is well documented, and the reality is that colposcopy is practiced by a wide range of clinicians. Ultimately, the combination of new technology with appropriate teaching of colposcopy and continuous quality improvement and evaluation of colposcopists is of outmost importance for the success of the cervical cancer screening process and ultimately for the life of the women.
